# To Stent or Not to Stent: A Tale of Two Occlusions

**DOI:** 10.7759/cureus.1364

**Published:** 2017-06-17

**Authors:** Ali S Haider, Lyndon K Lee, Tijani Osumah, Saira Alli, Umair Khan, Steven Vayalumkal, Aida Kafai Golahmadi, Richa Thakur, Phu Nguyen, Kennith F Layton

**Affiliations:** 1 Department of Neurosurgery, Scott and White Hospital, Temple, TX; 2 Texas A&M College of Medicine; 3 School of Medicine, Ross University; 4 Department of Neurosurgery, Leeds General Infirmary; 5 School of Medicine, St. Georges University; 6 School of Medicine, St. George's University; 7 Neurosurgery, Barts and the Royal London Hospitals; 8 Department of Radiology, Baylor University Medical Center

**Keywords:** acute ischemic stroke, thrombolysis, angioplasty, recanalization

## Abstract

Stenting and balloon angioplasty, along with mechanical thrombectomy, have gained notability as adjunctive treatment options to intravenous tissue plasminogen activator (IV-tPA) for tandem internal carotid artery (ICA) and middle cerebral artery (MCA) occlusions (TIM occlusions). Acute ischemic strokes (AISs) secondary to TIM occlusions are associated with poor patient outcomes primarily due to low recanalization rates following intravenous thrombolysis, consequently prompting the need for more invasive recanalization efforts. Often, the treatment algorithm is based on the success of the initial angioplasty, suspected volume of completed infarction, and whether or not thrombolytics are utilized. Here, we present two patients with AIS due to TIM occlusions where two different treatment modalities were implemented for recanalization efforts. Patient 1 did not receive IV-tPA and was successfully managed with balloon angioplasty and subsequent carotid stenting followed by direct oral anticoagulant (DOAC) administration. Patient 2 received IV-tPA and balloon angioplasty without carotid stenting followed by intracranial mechanical thrombectomy. Complete recanalization was attained in both cases. Administration of IV-tPA can make subsequent carotid stenting a potentially higher-risk treatment option for patients with TIM due to potential hemorrhagic complications in the setting of requisite antiplatelet agents. Each case of AIS resulting from a TIM must be considered unique, and the use of IV thrombolytics, balloon angioplasty, carotid stenting, and mechanical thrombectomy alone or in combination must be tailored to the individual clinical parameters.

## Introduction

Acute ischemic strokes (AISs) caused by tandem extracranial internal carotid artery (ICA) and middle cerebral artery (MCA) occlusions (TIM occlusions) are associated with poor short and long-term outcomes as they are often resistant to intravenous tissue plasminogen activator (IV-tPA)-based thrombolytic therapy [1-2]. Stenting of the extracranial ICA, coupled with intracranial thrombectomy, has emerged as a feasible alternative to traditional IV thrombolysis for treating tandem ICA/MCA occlusions [3-6]. However, carotid stenting is accompanied by the administration of antiplatelet medications that can raise the risk of symptomatic intracranial hemorrhage (SICH) in conjunction with thrombolytics [6-7]. The use of balloon angioplasty and mechanical thrombectomy after tPA administration has also been shown to yield beneficial results as they avoid the need for stent-associated antiplatelet therapy [8]. Thus, it is important to consider whether the patient received anticoagulation or thrombolytic agents when determining the necessity of stent placement. Here, we present two back-to-back cases of AIS on the same day resulting from TIM occlusion that were treated with different management strategies due to the surrounding circumstances in each scenario.

## Case presentation

### Patient 1

**Figure 1:**
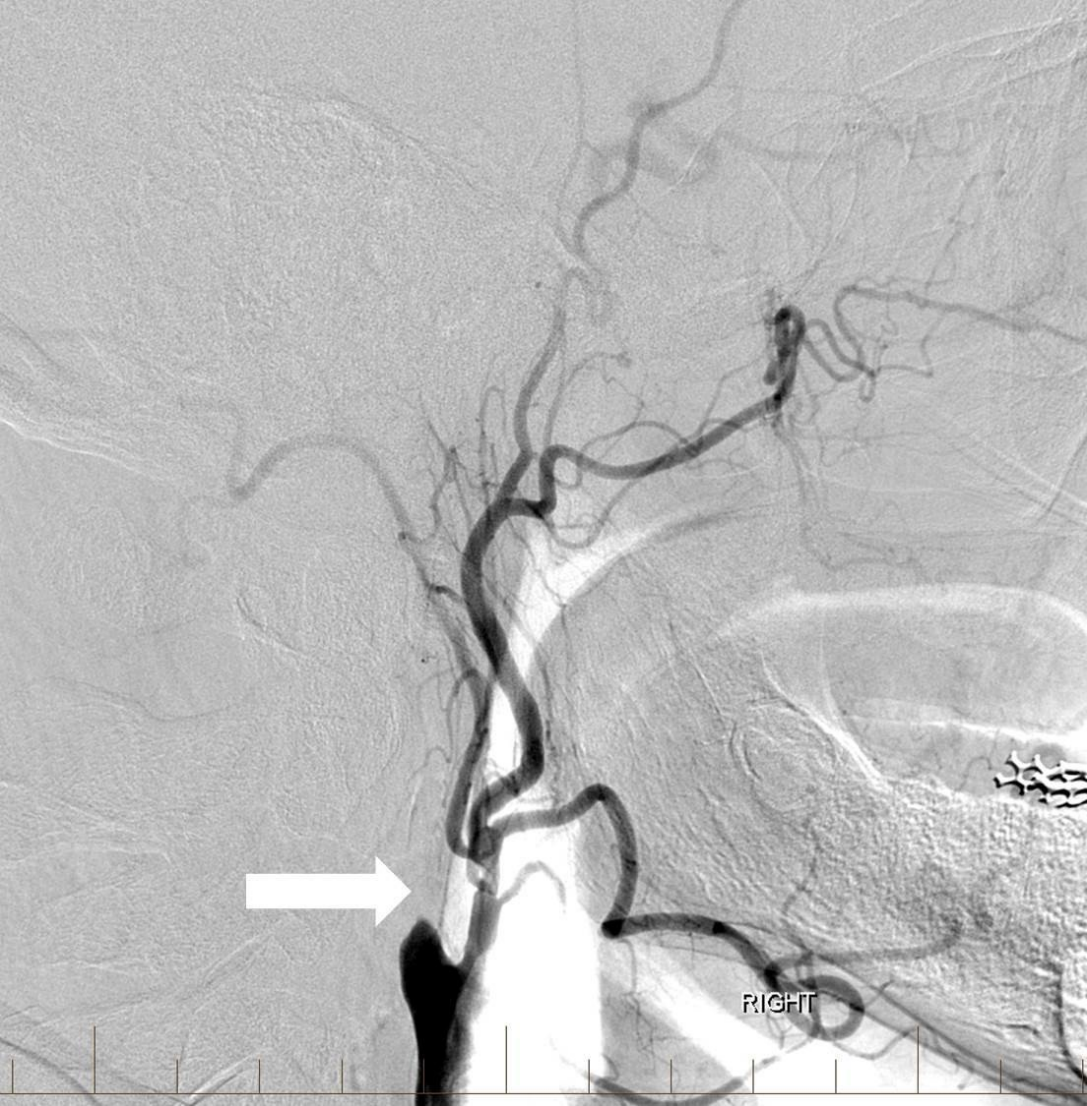
Lateral digital subtraction angiography image of the right common carotid artery showing complete occlusion of the extracranial internal carotid artery (arrow).

A 63-year-old man with a history of hypertension, hyperlipidemia, peripheral artery disease, smoking, and Stage IV lung cancer presented with left-sided weakness and dysarthria when he visited our institution for a chemotherapy port placement. His National Institutes of Health Stroke Scale (NIHSS) score was six and he was not a candidate for intravenous (IV) thrombolytics. Computed tomography angiography (CTA) revealed occlusion of the extracranial right internal carotid artery (ICA) and right middle cerebral artery (MCA) but no hemorrhage or large areas of acute ischemic change. Biplane digital subtraction angiography (DSA) revealed tapering to occlusion of the proximal right cervical ICA at the level of the carotid artery bulb (Figure 1).

**Figure 2:**
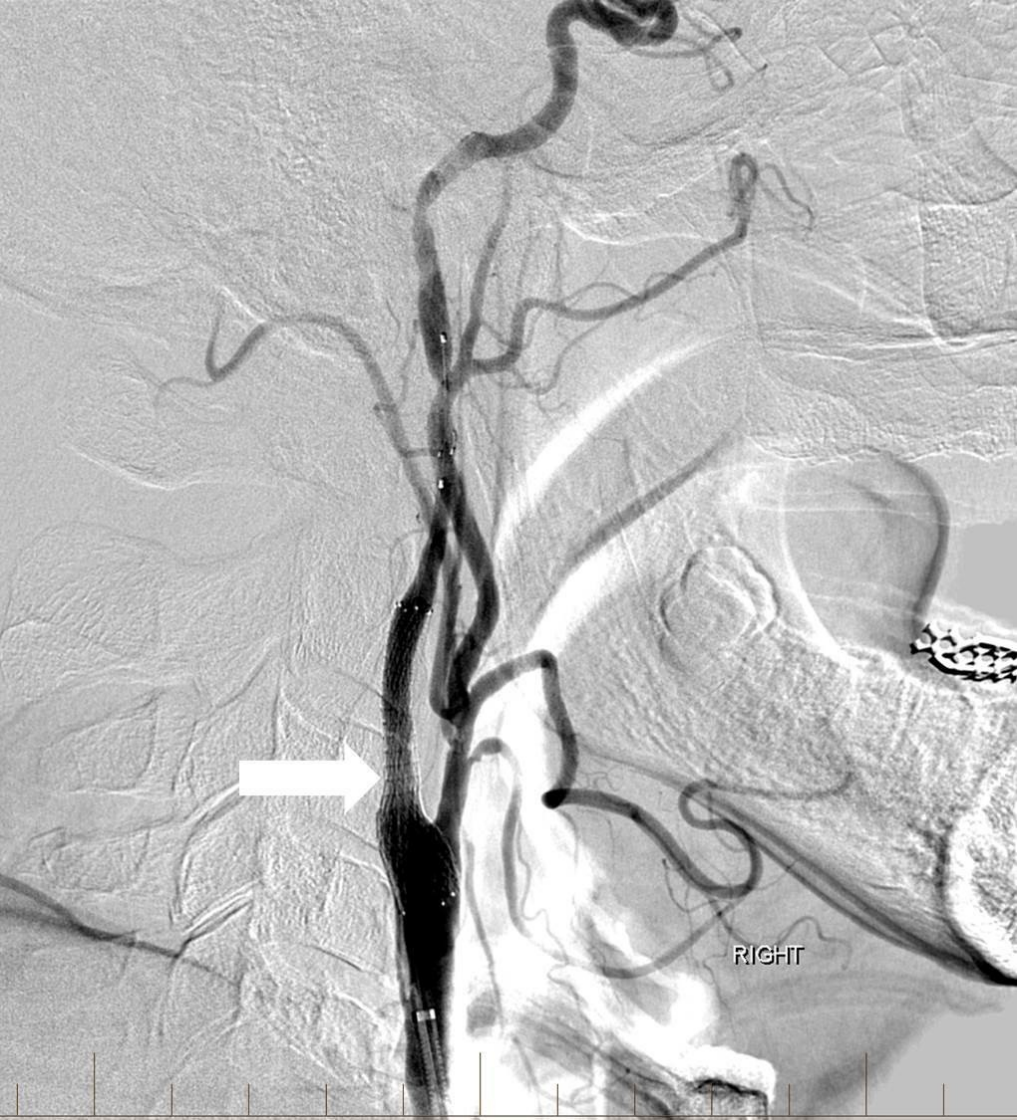
Lateral digital subtraction angiography image after balloon angioplasty and stenting with a distal protection device The right internal carotid artery is widely patent and there is no significant residual stenosis (arrow). "Right" indicates the patient's right side.

Angioplasty was performed at the level of the severe proximal ICA occlusion using a 4 mm x 20 mm noncompliant balloon. Subsequent angiography demonstrated the progression of contrast into the distal cervical and intracranial segments of the ICA with a persistent severe and irregular stenosis. A decision was then made to stent using distal embolic protection. A 4-mm diameter distal protection device was advanced and deployed in standard fashion within the distal cervical ICA at the level of C1-C2. An eptifibatide infusion was started, and an 8 mm to 6 mm tapered self-expanding carotid stent measuring 40 mm in length was positioned across the lesion and deployed in the standard fashion without angiographic complications. Post-stenting angiography revealed a 90% improvement in the caliber of the occluded vessel without delay in transit time throughout all cervical and intracranial right ICA segments (Figure 2).

**Figure 3:**
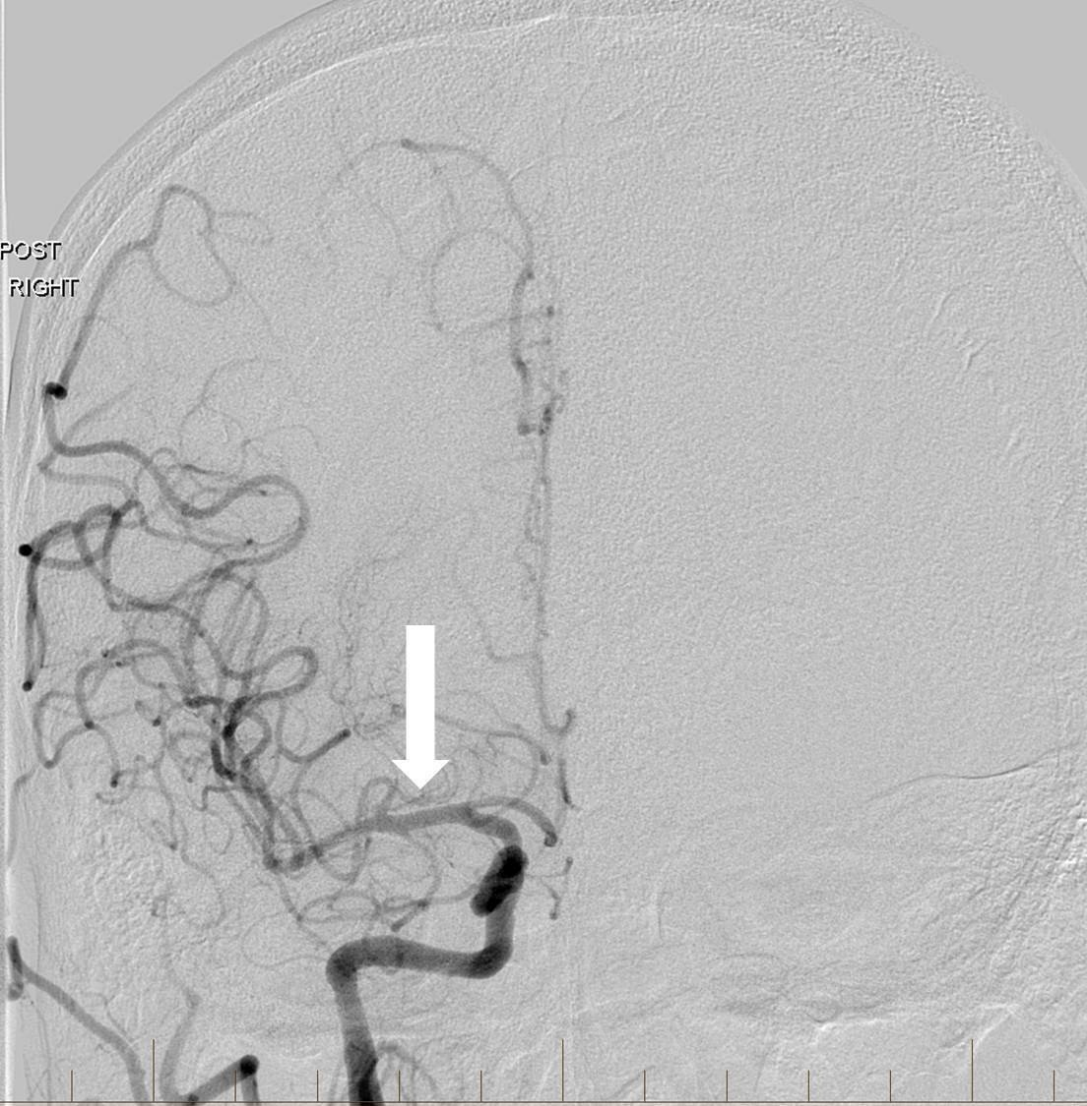
Frontal image of a right common carotid artery injection after angioplasty and stenting reveals recanalization of the right anterior circulation with normal intracranial flow (arrow). "Post" indicates the posterior aspect of the patient. "Right" designates the patient's right side.

The right MCA was then patent with excellent flow to the anterior circulation (Figure 3).

**Figure 4:**
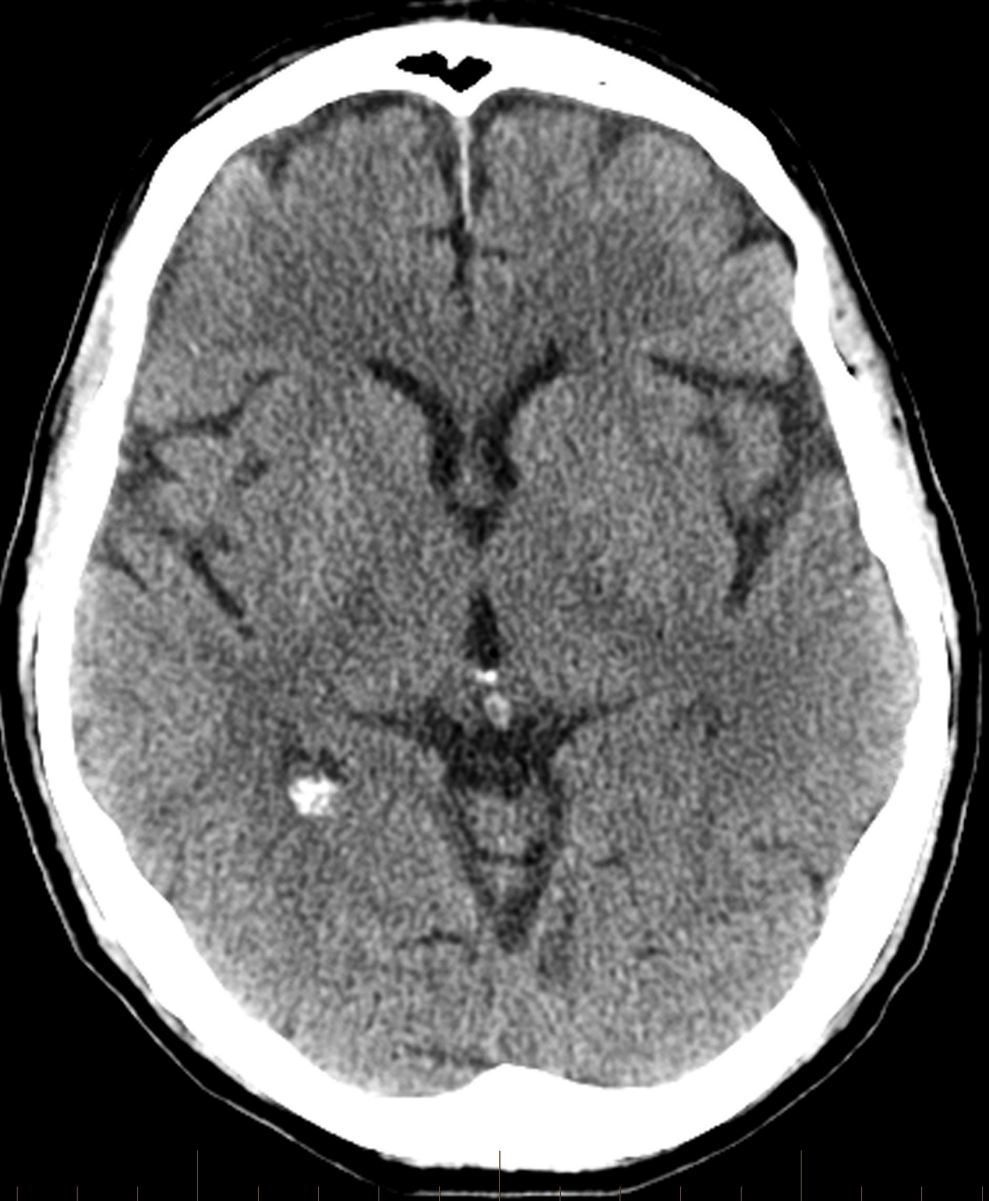
Follow-up head computed tomography performed early the next day shows no significant acute infarction or intracranial hemorrhage.

Follow-up head computed tomography (CT) revealed no hemorrhage or acute infarct (Figure 4).

The patient left the hospital the following day against medical advice. His NIHSS score prior to leaving had improved to two.

### Patient 2

**Figure 5:**
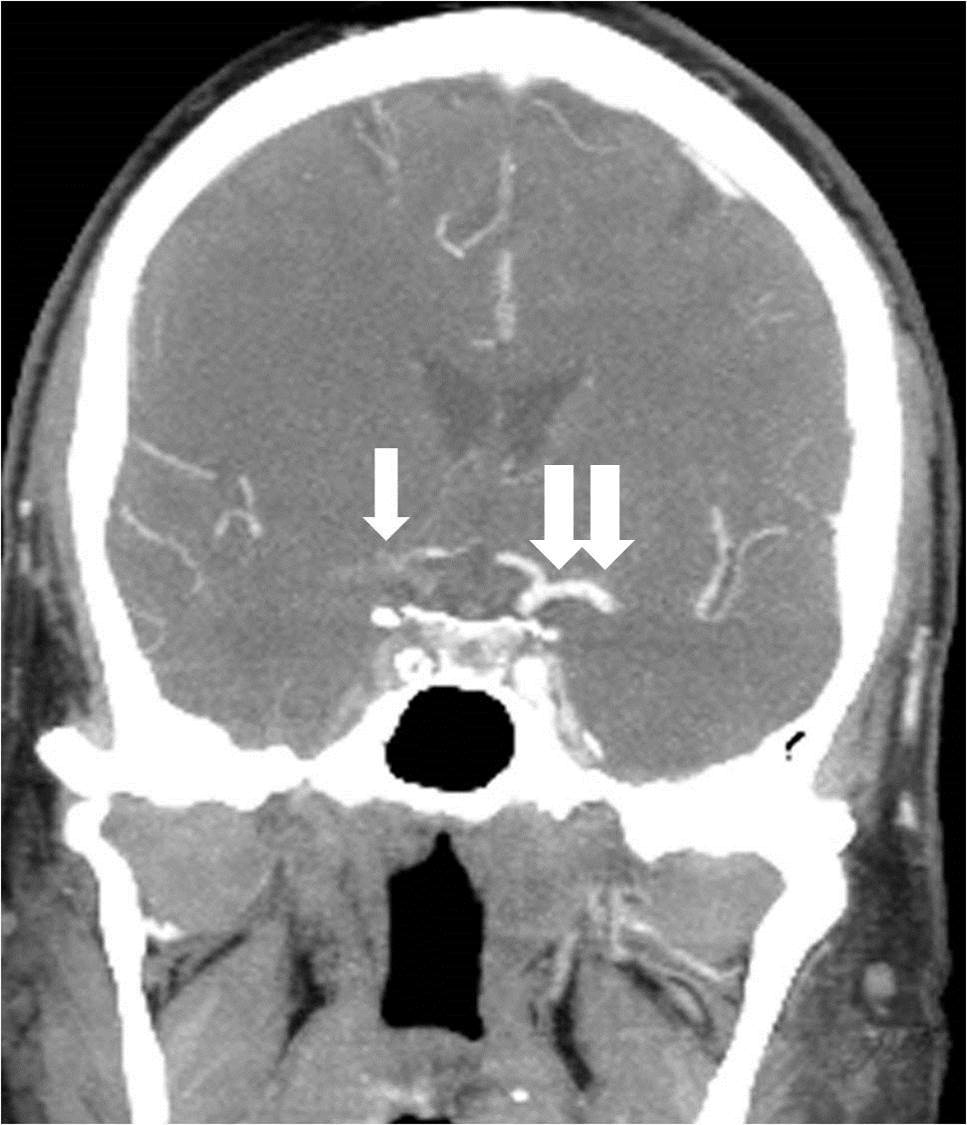
Coronal reconstruction from a computed tomography angiogram reveals a complete occlusion of the right middle cerebral artery and supraclinoid internal carotid artery (single arrow) Notice the normal contralateral middle cerebral artery and internal carotid artery (double arrow).

A 64-year-old man with a history of smoking and rheumatoid arthritis (RA) presented with left hemiparesis, slurred speech, and left facial droop. His NIHSS score was 9. Initial CTA showed occlusion of the extracranial right ICA and a tandem occlusion of the right MCA (Figure 5). 

**Figure 6:**
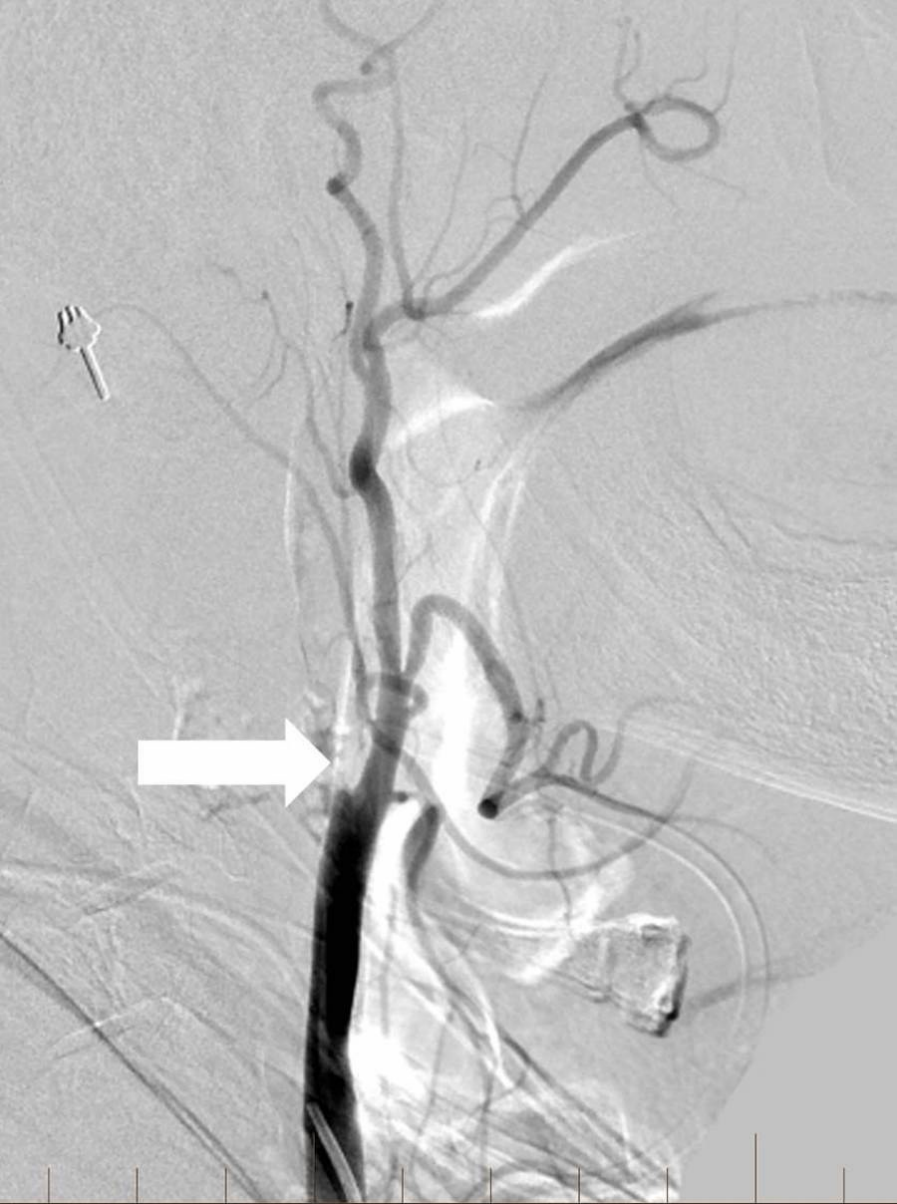
Lateral digital subtraction angiography image of the right common carotid artery shows complete occlusion of the extracranial internal carotid artery (arrow).

tPA was administered intravenously, and the patient was transferred to the interventional neuroradiology suite. Biplane digital subtraction angiography revealed a complete occlusion of the cervical right ICA at the vessel’s origin due to extensive atherosclerotic disease (Figure 6).

**Figure 7:**
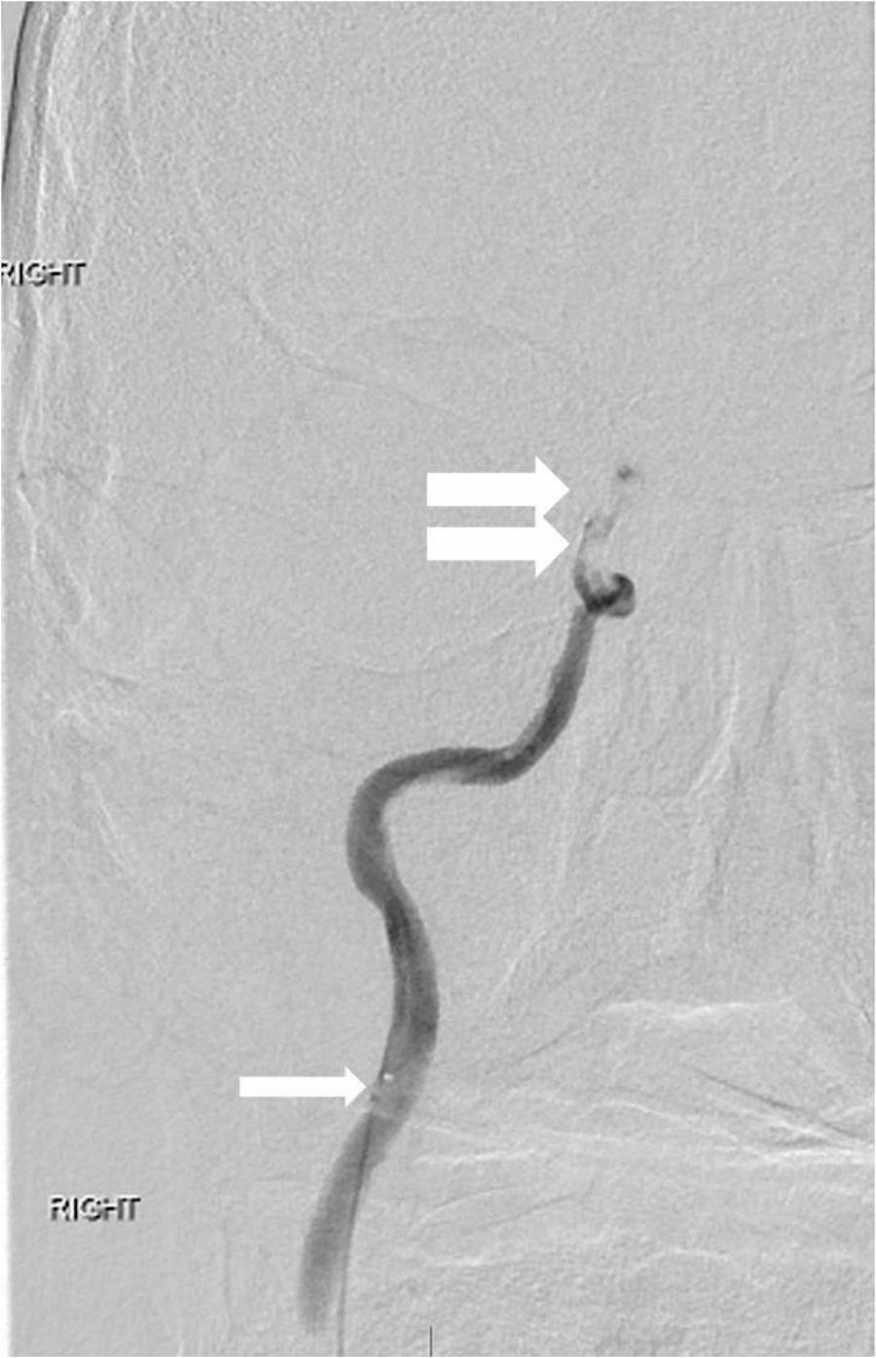
Anterior view right internal carotid artery injection through the microcatheter (single arrow) is performed beyond the occlusion There is a tandem occlusion of the distal internal carotid artery (double arrow) involving the intracranial internal carotid artery segments. "Right" designates the patient's right side.

The right MCA and right anterior cerebral arteries (ACAs) could not be visualized, and multifocal embolic filling defects were detected within the cavernous and supraclinoid ICA segments on microcatheter contrast injection past the carotid origin occlusion (Figure 7).

**Figure 8:**
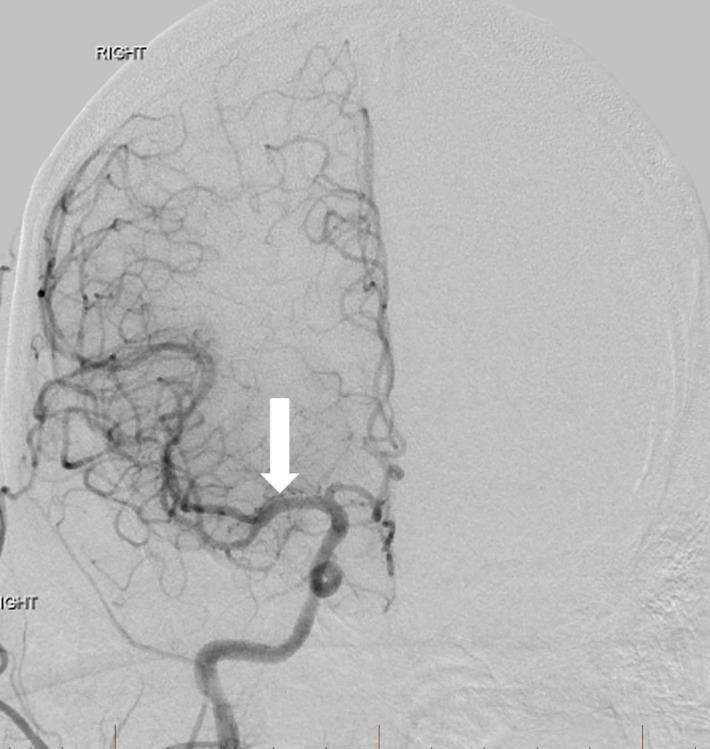
After angioplasty of the extracranial right internal carotid artery origin and intracranial thrombectomy, frontal view right common carotid artery angiogram reveals normal intracranial arterial flow with excellent recanalization (arrow). "Right" designates the patient's right side.

Given the previous administration of tPA, a decision was made to attempt balloon angioplasty without carotid stenting of the extracranial ICA. A 6 mm x 20 mm noncompliant balloon was positioned across the occluded proximal ICA and inflated until adequate luminal patency was restored. The thromboemboli in the supraclinoid ICA and MCA were subsequently removed by suction aspiration using an ACE 68 reperfusion catheter (Penumbra, Inc., Alameda, CA) and a direct aspiration first pass (ADAPT) technique [9]. Post-procedural right common carotid angiography demonstrated complete recanalization of the right middle cerebral and right anterior cerebral arteries without angiographic evidence of complicating features (Figure 8).

Angiography of the right common carotid artery demonstrated approximately a 40% residual stenosis at the ICA origin with no delay in the transit of contrast into the intracranial vasculature. A thrombolysis in cerebral infarction (TICI) score of three was achieved at the conclusion of the operation. Subsequent head computed tomography (CT) and magnetic resonance imaging (MRI) of the brain showed scattered infarcts in the right middle cerebral artery territory with multifocal petechial hemorrhages without frank hematoma. Compared to his initial NIHSS score of nine at presentation, he was discharged home three days later with an NIHSS of zero and no residual neurological deficits.

## Discussion

Up to one in six cases of AIS may involve TIM occlusion [1, 6]. Among patients with ICA occlusions, Christou, et al. found that nearly half had concurrent MCA occlusions, and intravenously administered tPA failed to recanalize the ICA in 74% of patients and the MCA in 41% of patients [10]. Rubiera, et al. demonstrated that TIM occlusions were strong predictors of poor outcomes after intravenous thrombolytic treatment with only a 41.9% recanalization success compared to 69.5% in patients with only MCA lesions [1]. Endovascular approaches have been established as effective revascularization therapies in these scenarios. A study conducted by Malik, et al. involving carotid stenting and various thromboembolus removal techniques achieved recanalization rate of 75.3% (thrombolysis in myocardial infarction score ≥ 2) [5]. Interestingly, 23% of the patients in the study experienced spontaneous intracranial revascularization immediately after the stent was placed, similar to Patient 1 in our report; this is likely due to improved inflow and use of antiplatelet agents during stenting [5]. Stampfl, et al. employed carotid stenting and stent retriever thrombectomy and reported a 62.5% recanalization (TICI ≥ 2b) rate among patients with tandem ICA/MCA occlusions [6]. The effect of periprocedural anticoagulant, antiplatelet, or thrombolytic therapy, particularly tPA, on the prevalence of SICH in endovascularly treated TIM cases is currently unclear. 

## Conclusions

Endovascular treatment modalities have been shown to achieve considerably significant rates of recanalization of TIM occlusions. Compared to IV thrombolytic therapy alone, the data favoring endovascular options continues to rise; endovascular methods have proven to be safe, effective alternatives with increasing favorability. With recanalization rates of TIM occlusions by IV thrombolysis remaining dismal, continued study of neurointerventional therapies and procedures is warranted. Comprehensive comparative and efficacy studies of specific endovascular therapies, such as balloon angioplasty, stenting, and mechanical thrombectomy, through long-term robust investigation will provide greater insight into individualized acute management options for AIS secondary to TIM. 

## References

[1] Rubiera M, Ribo M, Delgado-Mederos R (2006). Tandem internal carotid artery/middle cerebral artery occlusion: an independent predictor of poor outcome after systemic thrombolysis. Stroke.

[2] Kim YS, Garami Z, Mikulik R (2005). Early recanalization rates and clinical outcomes in patients with tandem internal carotid artery/middle cerebral artery occlusion and isolated middle cerebral artery occlusion. Stroke.

[3] Cohen JE, Gomori JM, Rajz G (2015). Extracranial carotid artery stenting followed by intracranial stent-based thrombectomy for acute tandem occlusive disease. J Neurointerv Surg.

[4] Mishra A, Stockley H, Goddard T (2015). Emergent extracranial internal carotid artery stenting and mechanical thrombectomy in acute ischaemic stroke. Interv Neuroradiol.

[5] Malik AM, Vora NA, Lin R (2011). Endovascular Treatment of Tandem Extracranial/Intracranial Anterior Circulation Occlusions: Preliminary Single-Center Experience. Stroke.

[6] Stampfl S, Ringleb PA, Möhlenbruch M (2014). Emergency cervical internal carotid artery stenting in combination with intracranial thrombectomy in acute stroke. AJNR Am J Neuroradiol.

[7] Heck DV, Brown MD (2015). Carotid stenting and intracranial thrombectomy for treatment of acute stroke due to tandem occlusions with aggressive antiplatelet therapy may be associated with a high incidence of intracranial hemorrhage. J Neurointerv Surg.

[8] Dababneh H, Guerrero WR, Khanna A (2012). Management of tandem occlusion stroke with endovascular therapy. Neurosurg Focus.

[9] Turk AS, Spiotta A, Frei D (2014). Initial clinical experience with the ADAPT technique: A direct aspiration first pass technique for stroke thrombectomy. J Neurointerv Surg.

[10] Christou I, Felberg RA, Demchuk AM (2002). Intravenous tissue plasminogen activator and flow improvement in acute ischemic stroke patients with internal carotid artery occlusion. J Neuroimaging.

